# Inflammation and depression: an evolutionary framework for the role of physical activity and exercise

**DOI:** 10.3389/fpsyg.2025.1554062

**Published:** 2025-05-29

**Authors:** Pedro Carrera-Bastos, Breno Bottino, Matthew Stults-Kolehmainen, Felipe Barreto Schuch, Fernando Mata-Ordoñez, Paulo T. Müller, José-Ramón Blanco, Daniel Boullosa

**Affiliations:** ^1^Faculty of Biomedical and Health Sciences, Universidad Europea de Madrid, Madrid, Spain; ^2^Center for Primary Health Care Research, Department of Clinical Sciences, Lund University, Malmö, Sweden; ^3^Rio Maior School of Sport - Santarém Polytechnic University, Rio Maior, Portugal; ^4^Federal University of Mato Grosso do Sul, Campo Grande, Brazil; ^5^Division of Digestive Health, Yale New Haven Hospital, New Haven, CT, United States; ^6^Department of Biobehavioral Sciences, Teachers College – Columbia University, New York, NY, United States; ^7^Department of Sports Methods and Techniques, Federal University of Santa Maria, Santa Maria, Brazil; ^8^Institute of Psychiatry, Federal University of Rio de Janeiro, Rio de Janeiro, Brazil; ^9^Faculty of Health Sciences, Universidad Autónoma de Chile, Providência, Chile; ^10^Maimonides Biomedical Research Institute of Cordoba (IMIBIC), Cordoba, Spain; ^11^Department of Cell Biology, Physiology, and Immunology, University of Cordoba, Cordoba, Spain; ^12^Reina Sofia University Hospital (HURS), Cordoba, Spain; ^13^Faculty of Health Sciences, Alfonso X el Sabio University, Villanueva de la Cañada, Spain; ^14^School of Medicine, Federal University of Mato Grosso do Sul, Campo Grande, Brazil; ^15^Infectious Disease Service, Hospital Universitario San Pedro, Logroño, La Rioja, Spain; ^16^La Rioja Center for Biomedical Investigation, Logroño, La Rioja, Spain; ^17^Faculty of Physical Activity and Sports Sciences, Universidad de León, León, Spain; ^18^Integrated Institute of Health, Federal University of Mato Grosso do Sul, Campo Grande, Brazil; ^19^College of Healthcare Sciences, James Cook University, Townsville, VIC, Australia

**Keywords:** Major Depressive Disorder, systemic low-grade chronic inflammation, neuroinflammation, mismatch, exercise, lifestyle

## Abstract

Major Depressive Disorder (MDD) is a leading global health challenge, affecting nearly 5% of the population. Mounting evidence suggests that systemic low-grade chronic inflammation (SLGCI) plays a central role in the development and progression of MDD. This persistent inflammatory state results from unresolved immune activation and sustained exposure to modern lifestyle factors, such as sedentary behavior, poor diet, inadequate sleep, and psychological stress. Regular physical activity (PA), particularly exercise, has been shown to modulate inflammatory processes and improve depressive symptoms. This narrative review examines the complex interactions between inflammation and MDD, focusing on the role of PA and exercise in mitigating SLGCI and neuroinflammation. This is approached through an evolutionary lens, exploring how the mismatch between ancestral and modern activity levels may contribute to the rise of MDD. In addition, it highlights the potential risks of excessive exercise, including overtraining and its association with depressive symptoms. Finally, this work proposes a practical framework for optimizing PA and exercise as preventive and therapeutic tools for MDD by aligning modern PA patterns with ancestral behavioral norms.

## 1 Introduction

Major Depressive Disorder (MDD) is a significant public health concern, affecting nearly 5% of the population worldwide (Malhi and Mann, 2018). Alarmingly, its prevalence appears to be rising, with several studies reporting increasing incidence rates over the past decades (GBD 2019 Mental Disorders Collaborators, 2022; Goodwin et al., 2022). While MDD is more prevalent in women (Li S. et al., 2023), it affects individuals across sexes (GBD 2019 Mental Disorders Collaborators, 2022) and age groups (Ghandour et al., 2019; Juul et al., 2021; GBD 2019 Mental Disorders Collaborators, 2022). Although various psychological and environmental factors are important in the etiology of MDD, growing research points to the central involvement of biological mechanisms in both its onset and progression (Miller and Raison, 2016; Malhi and Mann, 2018; Correia et al., 2023; Hassamal, 2023). These mechanisms include neurotransmitter dysregulation, altered hypothalamic–pituitary–adrenal axis function, impaired neuroplasticity, oxidative stress, and notably, chronic inflammation (Miller and Raison, 2016; Correia et al., 2023; Hassamal, 2023).

Regarding inflammation, it serves as an evolutionarily conserved mechanism essential for host protection and the restoration of homeostasis (Medzhitov, 2021; Meizlish et al., 2021). Under normal circumstances, it typically resolves once these functions are achieved (Furman et al., 2019). However, when inflammatory responses fail to resolve—due to intrinsic dysregulation or persistent exposure to stressors—they may evolve into a state of systemic low-grade chronic inflammation (SLGCI) (Furman et al., 2019). SLGCI is now recognized as a shared mechanism underlying several chronic diseases, such as cancer, autoimmune diseases, non-alcoholic fatty liver disease, type 2 diabetes, cardiovascular disease (CVD), chronic kidney disease, osteoporosis, sarcopenia, neurodegenerative diseases, and psychiatric disorders such as MDD (Haapakoski et al., 2015; Köhler et al., 2017; Arteaga-Henríquez et al., 2019; Furman et al., 2019; Osimo et al., 2019; Bai et al., 2020; Costanza et al., 2024; Vöckel et al., 2024; Yin et al., 2024).

A variety of intrinsic and extrinsic modifiable factors contribute to SLGCI, including smoking, environmental pollutants, psychological stress, sleep disturbances and circadian disruption, poor diets, excessive adiposity, and physical inactivity (Furman et al., 2019; Burini et al., 2020; Valenzuela et al., 2023). Interestingly, the advent of most of these stressors postdates the Neolithic and, particularly, the Industrial Revolution, representing a relatively short period on the evolutionary timeline for human physiology to fully adapt (Cordain et al., 2005; Carrera-Bastos et al., 2011; Ruiz-Núñez et al., 2013; Burini et al., 2020; Chaudhary and Salali, 2022). It can, therefore, be argued that the rapid emergence of various ‘diseases of civilization’—including MDD—may reflect a mismatch between our ancestral physiology and modern lifestyles (Carrera-Bastos et al., 2011; Ruiz-Núñez et al., 2013; Burini et al., 2020; Chaudhary and Salali, 2022). Among these modifiable factors, sedentary behavior and physical inactivity stand out due to their widespread prevalence (Guthold et al., 2018, 2020) and its numerous pleiotropic effects (Burini et al., 2020; Kerr and Booth, 2022; Pinto et al., 2023).

From an evolutionary perspective, most of the human genome evolved under conditions characterized by high physical activity (PA) (Cordain et al., 1998; Boullosa et al., 2013; Booth et al., 2017). Virtually all hominins, including *Homo sapiens*—which arose approximately 200,000–300,000 years ago (Hublin et al., 2017; Richter et al., 2017; Schlebusch et al., 2017; Vidal et al., 2022)—depended on PA for survival, i.e., hunting and gathering, fleeing predators, digging, carrying loads, and other tasks involving both low- and high-intensity physical activities (Boullosa et al., 2013; Booth et al., 2017). However, after the Industrial Revolution and the advent of the Modern era, drastic changes in lifestyle occurred, reducing the need for PA at any intensity and increasing sedentary behavior (Eaton and Eaton, 2003). These lifestyle changes may have a pivotal role in contributing to diseases of civilization, such as MDD (Booth et al., 2017). Conversely, as will be discussed in subsequent sections, there is extensive evidence supporting the role of high levels of PA and regular exercise in reducing inflammation, as well as in preventing and ameliorating MDD. Notably, while pharmacological and psychotherapeutic approaches remain the mainstay of MDD treatment, current evidence suggests that exercise has antidepressant effects that are, in magnitude, comparable to traditional MDD therapies (Fabiano et al., 2025). Nevertheless, while these effect sizes are similar, exercise should not be seen as a replacement for conventional interventions but as an adjunctive therapeutic strategy (Fabiano et al., 2025). Interestingly, the mental health benefits of exercise appear to follow an inverted U-shaped curve, with very high levels of PA—exhibited typically by athletes, manual laborers, and individuals with exercise dependence—being associated with depressive-like symptoms (Armstrong and VanHeest, 2002; Gouttebarge et al., 2019; Golding et al., 2020; Golshani et al., 2021).

This narrative review explores how evolutionary insights can guide the use of PA and exercise in MDD prevention and treatment. We examine the interplay between inflammation and depression, the dual-edged nature of exercise, and how ancestral activity patterns may inform optimal PA prescriptions in modern settings.

## 2 Inflammation and depression

As previously mentioned, inflammation is a biologically-essential process that protects the host from pathogens, toxins, and other insults, while also facilitating tissue repair and restoring homeostasis (Medzhitov, 2021; Meizlish et al., 2021). However, this process involves metabolic and neuroendocrine changes that, if left unchecked, can impair survival and reproductive capacity (Straub and Schradin, 2016). Therefore, under normal conditions, inflammation is a time-limited acute response that resolves upon achieving its protective goals (Furman et al., 2019). Nevertheless, failures in the resolution of inflammation—due to impaired anti-inflammatory signaling, insufficient clearance of apoptotic cells, defects in efferocytosis, or chronic exposure to inflammatory stimuli—can lead to persistent immune activation (Kourtzelis et al., 2020; Doran, 2022; Panezai and Van Dyke, 2022; Collins et al., 2023). This sustained, dysregulated state is referred to as SLGCI, characterized by mildly elevated circulating inflammatory biomarkers and associated with a range of chronic degenerative conditions (Furman et al., 2019), including MDD (Arteaga-Henríquez et al., 2019; Costanza et al., 2024; Yin et al., 2024).

From an evolutionary standpoint, inflammation is tied to the concept of sickness behavior—an adaptive response featuring lethargy, anhedonia, social withdrawal, and reduced appetite, aimed at conserving energy to prioritize immune functions and recovery (Straub et al., 2010; Dooley et al., 2018). These behavioral and metabolic adaptations likely conferred survival advantages in ancestral environments where acute threats were common (Straub and Schradin, 2016). However, in modern contexts marked by persistent stressors and lower pathogen exposure, these once protective pathways can drive SLGCI, contributing to the onset and progression of MDD (Miller and Raison, 2016). In this sense, SLGCI does not appear to be a normal or expected physiological state from an evolutionary perspective. Human physiology seems to have evolved to cope with acute, well-regulated inflammatory responses—not with persistent, low-grade inflammation (Furman et al., 2019; McDade, 2023). Supporting this notion, studies in traditional and non-industrialized populations—including the Melanesian horticulturalists of Kitava in Papua New Guinea (Carrera-Bastos et al., 2020), subsistence-agriculturalists in rural Ghana (Eriksson et al., 2013), the Shuar forager-horticulturalists of the Ecuadorian Amazon (McDade et al., 2012), and rural Filipinos (McDade, 2023)—have consistently documented extremely low baseline levels of C-reactive protein (CRP), despite frequent exposure to infectious agents and limited access to modern sanitation or medical care. These findings suggest that the low-grade chronic inflammatory state commonly observed in industrialized societies likely reflects a mismatch between modern environments and our evolutionary heritage.

### 2.1 Neuroinflammation

Neuroinflammation, a localized inflammatory response within the central nervous system (CNS), appears to be an important mechanism linking chronic inflammation to MDD. It is primarily mediated by glial cells, especially microglia—the brain’s resident immune cells (Yang and Zhou, 2019; Li et al., 2024). Upon activation by stress, trauma, or peripheral inflammatory signals resulting from SLGCI, microglia adopt a pro-inflammatory phenotype, releasing inflammatory cytokines, such as tumor necrosis factor-α (TNF-α), interleukin-1β (IL-1β), and interleukin-6 (IL-6) (Li et al., 2024). These molecules can disrupt neuronal communication and impair neuroplasticity (Li et al., 2024; Pape et al., 2019). Moreover, they activate endothelial cells of the blood–brain barrier (BBB), increasing its permeability and facilitating the infiltration of peripheral immune cells—including monocytes, neutrophils, and T-cells—into the CNS (Felger, 2018; Lee and Giuliani, 2019; Beurel et al., 2020). This amplifies neuroinflammation by increasing the burden of pro-inflammatory molecules and further activating glial cells, which release reactive oxygen species (ROS) and nitric oxide (Beurel et al., 2020; Serafini et al., 2023; Kouba et al., 2024; Yin et al., 2024). The resulting oxidative and nitrosative stress impairs synaptic plasticity and disrupts the fronto-limbic network, which is critical for mood regulation (Kouba et al., 2024; Maas et al., 2017; Han and Ham, 2021).

Pro-inflammatory cytokines also affect neurotransmitter metabolism through activation of indoleamine 2,3-dioxygenase (IDO) (Seo and Kwon, 2023; Yin et al., 2024), which diverts tryptophan from serotonin synthesis toward kynurenine production (Seo and Kwon, 2023). Kynurenine is metabolized into neurotoxic compounds, such as quinolinic acid, which activate N-methyl-D-aspartate (NMDA) receptors and promote glutamate excitotoxicity (Kouba et al., 2024; Yin et al., 2024). This sequence of events impairs neuroplasticity, reduces hippocampal neurogenesis, and disrupts serotonin and dopamine signaling, contributing to anhedonia, motivational deficits, and other core symptoms of MDD (Cui et al., 2024). Additionally, chronic inflammation reduces levels of brain-derived neurotrophic factor (BDNF) (Yap et al., 2021), a key modulator of synaptic plasticity and neuronal resilience (Xiong et al., 2024), further impairing brain areas central to mood regulation, including the prefrontal cortex, amygdala, and hippocampus (Poletti et al., 2024).

### 2.2 Evidence linking chronic inflammation to depression

Multiple lines of evidence support the role of SLGCI in MDD. In animal models, systemic inflammatory triggers (e.g., endotoxins, cytokines) induce depressive-like behaviors and disrupt neurotransmitter regulation (Remus and Dantzer, 2016; Rhie et al., 2020; Yin et al., 2023). These models have elucidated specific inflammatory pathways involved in CNS dysfunction, such as the activation of IDO and its downstream effects. Epidemiological studies have consistently shown that elevated levels of inflammatory biomarkers are associated with increased depression risk (Smith et al., 2018; Li X. et al., 2023; Ji et al., 2024). Mendelian randomization analyses provide additional evidence by demonstrating a causal link between genetically predicted elevations in inflammatory biomarkers—such as CRP and IL-6—and increased susceptibility to depression (Khandaker et al., 2020).

Further support comes from meta-analyses and systematic reviews showing that patients with MDD exhibit higher concentrations of inflammatory biomarkers (Haapakoski et al., 2015; Köhler et al., 2017; Osimo et al., 2019; Li X. et al., 2023), which are also predictive of poor response to pharmacological antidepressant treatment (Arteaga-Henríquez et al., 2019). Moreover, these biomarkers are associated, in multiple epidemiological studies, with increased cardiovascular risk (Ridker, 2016; Li Y. et al., 2017; Li H. et al., 2017; Ni et al., 2020; Georgakis et al., 2021; Ridker et al., 2023, 2024a, 2024b; Khan et al., 2024), reinforcing the shared inflammatory underpinnings of MDD and CVD. In fact, recent meta-analytical evidence has associated MDD with a higher risk of cardiovascular mortality (Krittanawong et al., 2023).

Nevertheless, the most compelling evidence for the role of chronic inflammation in the etiology of MDD comes from randomized controlled trials (RCTs), which demonstrate the efficacy of anti-inflammatory agents—such as cytokine antagonists, nonsteroidal anti-inflammatory drugs, and omega-3 fatty acids—in reducing depressive symptoms in patients with MDD (Bai et al., 2020; Vöckel et al., 2024). Collectively, these findings support the role of inflammation as both a contributor to and a potential therapeutic target in MDD.

## 3 Physical activity, depression and inflammation

The inverse association between PA and MDD is supported by extensive epidemiological and clinical evidence. Individuals with depression consistently report lower PA levels and are approximately 50% less likely to meet public health guidelines recommending 150 min per week of moderate-to-vigorous PA compared to their age- and sex-matched peers (Schuch et al., 2017). In fact, they spend less time in all intensities of PA and more time in sedentary behavior (Schuch et al., 2017). Conversely, greater engagement in leisure-time PA is consistently associated with a lower risk of incident depression (Schuch et al., 2018; Werneck et al., 2023). Longitudinal studies, including those spanning several years, confirm these relationships (Schuch et al., 2018). In individuals already diagnosed with MDD, even acute bouts of exercise have been shown to enhance mood and increase feelings of vigor and wellbeing (Bourke et al., 2022).

Exercise interventions—a structured subset of PA designed to improve or sustain one or more physical fitness valences, such as muscular strength or cardiorespiratory capacity—have consistently shown efficacy in reducing depressive symptoms in individuals with either clinical or subclinical depression (Heissel et al., 2023). A recent systematic review and meta-analysis of RCTs by Heissel et al. concluded that both endurance training (also known as aerobic exercise) and resistance training (also referred to as strength or weight training) produce moderate-to-large effect sizes (Heissel et al., 2023). Moreover, higher-intensity exercise interventions were associated with greater reductions in depressive symptoms than lower-intensity protocols (Heissel et al., 2023). These findings apply to both aerobic and resistance exercise modes. The main results from the Heissel et al. meta-analysis are summarized in Table 1. At the population level, prospective cohort studies have also shown an inverse curvilinear association between PA and depression risk: complete physical inactivity corresponds to the highest incidence of depression, while the risk steadily decreases as PA levels increase (Pearce et al., 2022). This graded pattern may partially reflect the cumulative physiological benefits of increased PA levels and regular exercise, including its capacity to modulate inflammation and enhance neurobiological resilience.

**Table 1:** Effects of exercise on depressive symptoms: subgroup analyses by intensity and type.

Analysis	Number of RCTs	Meta analysis			
		SMD	95%CI		*p*-value
**Main analysis**	41	−0.946	−1.179	−0.714	**<0.001**
**Exercise intensity**					
Light	2	−1.041	−2.528	0.445	0.170
Moderate	26	−1.132	−1.453	−0.811	**<0.001**
Vigorous	10	−0.924	−1.472	−0.376	**0.001**
**Exercise type**					
Aerobic	30	−1.156	−1.461	−0.850	**<0.001**
Resistance	7	−1.042	−1.865	−0.218	**0.013**
Mixed^c^	10	−0.455	−0.797	−0.113	**0.009**

Summary of results from the systematic review and meta-analysis by Heissel et al. (2023), examining the effects of exercise interventions on depressive symptoms across different intensities and types. The analysis demonstrates that moderate and vigorous intensity exercise are associated with significant reductions in depressive symptoms, with aerobic and resistance training showing the largest effect sizes (Heissel et al., 2023).

Bold p-values indicate statistically significant results (*p* < 0.05). c refers to mixed exercise interventions combining aerobic and resistance modalities.

RCT, Randomized Controlled Trial; SMD, Standardized Mean Difference; CI, Confidence Interval.

Despite the robust antidepressant effects of exercise, the underlying neurobiological mechanisms remain incompletely understood (Schuch et al., 2016; Stubbs and Schuch, 2019; Sun W. et al., 2023). A prominent hypothesis posits that exercise modulates several immune pathways, leading to long-term adaptations in the inflammatory response (Nieman and Wentz, 2019; Antunes et al., 2020; Scheffer and Latini, 2020; Sun S. et al., 2023; Langston and Mathis, 2024). Acute bouts of exercise initially elicit a transient pro-inflammatory response due to tissue stress and damage, especially in the cardiovascular and musculoskeletal systems (Cornish and Cordingley, 2024; Langston and Mathis, 2024). This response is required to clear cellular debris and facilitate tissue repair, thus leading to morphological adaptations (Langston and Mathis, 2024). Subsequently, an anti-inflammatory cascade is activated to restore homeostasis and promote the resolution of acute inflammation (Beiter et al., 2015; Docherty et al., 2022; Langston and Mathis, 2024). With consistent exercise training, this biphasic response becomes more efficient, and exercise contributes to long-term reductions in inflammation—not only at the skeletal muscle level but across various organs and systems, including the CNS (Scheffer and Latini, 2020; Di Ludovico et al., 2024).

However, findings from studies specifically examining the effects of exercise on inflammatory biomarkers in individuals with MDD remain limited and sometimes conflicting. A recent meta-analysis of 10 studies investigating the inflammatory response to exercise in people with MDD found no significant acute effects of diverse exercise interventions on IL-6, IL-10, or IL-8. In contrast, chronic exercise was associated with a small but statistically significant increase in TNF-α levels (Standardized Mean Difference = 0.296; 0.03–0.562, *p* = 0.029), while no significant chronic effects were observed for IL-6 or IL-1β (Guimarães et al., 2024). These results must be interpreted with caution due to methodological heterogeneity, the confounding anti-inflammatory effects of antidepressants (Patel et al., 2023), and a high risk of bias across studies. Additional high-quality trials are needed to clarify these findings and further evaluate the role of inflammatory modulation in the antidepressant effects of exercise.

## 4 Excessive exercise, overtraining and depression-like conditions

While regular physical activity and exercise confer significant benefits for mental health, excessive exercise may paradoxically lead to adverse psychological outcomes, including depression-like symptoms or even clinical depression. In such cases, individuals often experience persistent fatigue, mood disturbances, and performance decrements that require extended recovery periods. This maladaptive state, known as overreaching, is commonly conceptualized as a continuum, ranging from “functional overreaching” (FOR) to “non-functional overreaching” (NFOR) and, ultimately, “overtraining syndrome” (OTS) (Kellmann et al., 2018; Brenner et al., 2024). FOR, when strategically incorporated into training cycles, temporarily impairs performance but may ultimately enhance fitness. However, recent evidence challenges the necessity of FOR, suggesting that it may not be required for performance enhancement and could even be detrimental to health (Bellinger, 2020). In contrast, NFOR reflects a failure of adaptation characterized by negative psychological and physical changes and persistent performance deficits (Kellmann et al., 2018).

With prolonged exercise stress, insufficient recovery, and the compounding effects of additional factors, such as background stress, poor sleep, and inadequate nutritional status (Stellingwerff et al., 2021), athletes may progress from NFOR to a state of staleness or even burnout. This more severe condition—often referred to as OTS—has been described as “athletes’ depression” (Raglin et al., 2000; Armstrong and VanHeest, 2002). Unfortunately, no unified definition exists for these terms, and there is a lack of consensus across the literature. Moreover, the bidirectional relationship between depression and PA (Roshanaei-Moghaddam et al., 2009) complicates the differentiation between causation and correlation. As a result, recent literature has emphasized the need for greater conceptual clarity and standardization in this field (Kellmann et al., 2018; Eklund and DeFreese, 2021; Madigan, 2021). In line with these challenges, there is increasing recognition that “sport burnout” shares many psychological and physiological features with clinical depression (Armstrong and VanHeest, 2002). Burnout in exercise and sport contexts is often associated with reduced enjoyment and pleasure during exercise (Nixdorf et al., 2023). More than two decades ago, Armstrong and VanHeest identified multiple similarities between OTS and depression, including depressed mood, lack of motivation, changes in body composition, insomnia, appetite disturbances, and feelings of irritability and restlessness (Armstrong and VanHeest, 2002). In a study involving high-level adolescent Swiss athletes, burnout scores were significantly correlated with depressive symptoms (*r* = 0.40) (Gerber et al., 2018). Similarly, very high levels of exercise have been linked to worse mental health outcomes (Chekroud et al., 2018). In elite athletes, Grasdalsmoen et al. (2022) reported that such negative outcomes were primarily observed in female athletes training more than 14 h per week. Collectively, these findings underscore the shared mechanisms between OTS and depression and highlight the importance of prevention strategies and individualized training protocols.

Burnout, NFOR, OTS, and depression all share conceptual and mechanistic roots within the paradigm of chronic stress (Kenttä and Hassmén, 1998; Nixdorf et al., 2023). Smith, as early as 1986, was among the first to explicitly define burnout as a maladaptive response to chronic stress exposure (Smith, 1986). However, the specific mechanisms by which prolonged stress leads to burnout and OTS remain complex and incompletely understood. One proposed mechanism is that chronic psychological stress can interfere with physical recovery following strenuous or high-intensity exercise, potentially leading to delayed recovery of muscular function and reduced physical performance (Stults-Kolehmainen and Bartholomew, 2012; Stults-Kolehmainen et al., 2014a). In addition, high levels of psychological and life stress are well-established risk factors for the onset of depression (Turner and Lloyd, 2004; Hammen, 2005; Stults-Kolehmainen et al., 2014b). Individuals exposed to both physical and psychological stressors display varying degrees of resilience, depending on factors such as mental health status, physical fitness, and social support. Nonetheless, each individual has a finite threshold beyond which accumulated stress can exceed adaptive capacity and trigger maladaptive outcomes.

According to the “resources versus demands” model of stress, athletes who encounter excessive physical or emotional demands without adequate recovery resources—such as rest, sleep, nutritional support, or social–emotional buffering—are at higher risk of burnout and are less able to sustain the demands of training and competition (Brenner et al., 2024). The consequences often include loss of enjoyment, declining motivation, overuse injuries, and eventual withdrawal from sport (Raglin et al., 2000; Meeusen et al., 2006; DiFiori et al., 2014). Dysregulation of inflammatory pathways has been proposed as a possible link between prolonged stress exposure and the development of depressive-like conditions in this context, though conclusive evidence is still lacking (Kim et al., 2022; Hassamal, 2023).

From an evolutionary perspective, it is plausible that prolonged exposure to excessive stressors triggers a shift toward energy-conserving states, manifesting as depression-like behaviors aimed at reducing further physical, psychological, or metabolic strain. Alternatively, stress-induced depression may represent a more fundamental biological strategy to preserve homeostasis by withdrawing from unsustainable environmental demands (Beck and Bredemeier, 2016).

## 5 An evolutionary approach to exercise as a treatment for MDD through the reduction of inflammation

Based on the current evidence, two complementary evolutionary perspectives can guide the use of PA and exercise in the prevention and treatment of MDD: (1) modeling patients’ PA patterns—including exercise—after those of ancestral human populations, and (2) selecting exercise modalities that specifically target SLGCI and neuroinflammation. Although exercise has demonstrated effects comparable to pharmacological and psychotherapeutic treatments for MDD (Fabiano et al., 2025), there is no consensus on the most effective types, intensities, or durations of exercise interventions (Heissel et al., 2023). By applying an evolutionary framework, exercise modalities can be selected not only for their anti-inflammatory properties but also for their compatibility with human physiology shaped by millennia of physically demanding lifestyles. This approach may provide broader physical and mental health benefits and extend to both prevention and treatment of MDD within a holistic, lifestyle-based perspective. In doing so, it may also enhance ecological validity and adherence to exercise-based interventions.

The available evidence supports a tentative recommendation for combining diverse forms of exercise within a context of reduced sedentary behavior—consistent with ancestral PA patterns. This would involve daily low-to-moderate PA interspersed with less frequent bouts of high-intensity activity (Boullosa et al., 2013). These activity levels exceed those typically observed in industrialized populations but are well within the physiological range of modern hunter-gatherer groups (Raichlen et al., 2017; Pontzer et al., 2018). This distinction is critical because excessive exercise—such as ultra-endurance training—has been associated with increased cardiovascular risk, potentially mediated by SLGCI (Celeski et al., 2024). Importantly, although individuals in both industrialized and ancestral societies may spend similar time resting, the latter use active resting postures (e.g., squatting), which promote greater muscle activation and favor musculoskeletal health (Raichlen et al., 2020).

Of note, the effectiveness of exercise interventions may be enhanced when performed in environments that optimize both mental and physical wellbeing—such as natural settings—and ideally involve social interactions with family or friends. These contexts have been shown to improve stress management (Antonelli et al., 2019; Bramwell et al., 2023), enhance enjoyment (Davis et al., 2021), and increase vitamin D levels via sun exposure (Wacker and Holick, 2013)—all factors associated with reduced depressive symptoms and systemic inflammation (Hansen et al., 2017; Gorman et al., 2019; Burns et al., 2021; Yeon et al., 2021; Kuczynski et al., 2022; Moslemi et al., 2022; Lin et al., 2023; Mikola et al., 2023; Siah et al., 2023; Wang et al., 2023).

When selecting exercise modalities to decrease SLGCI and neuroinflammation, two primary therapeutic targets emerge: (1) improvement in key physical fitness components—such as aerobic capacity and muscular strength—and (2) favorable changes in body composition. With respect to the first target, it is now well established that regular exercise elicits a cascade of physiological and molecular adaptations that directly counteract inflammatory processes. These include increased fluid shear stress, the release of exerkines (e.g., IL-6 with anti-inflammatory properties, BDNF), improved mitochondrial function, and modulation of both innate and adaptive immunity (Gleeson et al., 2011; Fiuza-Luces et al., 2013; Casuso and Huertas, 2021; Gao et al., 2024; Zhou et al., 2024; Chatzigeorgiou et al., 2025). Together, these adaptations may reduce peripheral and central inflammation (Hu et al., 2024), enhance neuroplasticity via neurogenesis, synaptogenesis, dendritic arborization, and angiogenesis (Morland et al., 2017; Lin et al., 2018; Xie et al., 2021), and alleviate depressive symptoms (Xie et al., 2021). These mechanisms offer a biological rationale for how improvements in aerobic capacity and muscular strength may help downregulate inflammatory activity and alleviate depressive symptoms. Supporting this, multiple controlled trials and systematic reviews have shown that both aerobic and resistance training—individually or in combination—can significantly reduce pro-inflammatory biomarkers (Fedewa et al., 2017; Bautmans et al., 2021; Kanthajan et al., 2024). Moreover, preliminary evidence suggests these modalities may also attenuate neuroinflammation (Hu et al., 2024), although more high-quality trials are needed to confirm these effects.

The second therapeutic target concerns body composition, with particular emphasis on reducing visceral adipose tissue (VAT), a depot known to play a central role in SLGCI (Valenzuela et al., 2023). Located within the abdominal cavity and surrounding internal organs, VAT is more metabolically active than subcutaneous fat and exhibits greater lipolytic activity (Hill et al., 2018; Cypess, 2022; Valenzuela et al., 2023). As VAT expands in the context of obesity, it becomes prone to hypoxia due to inadequate vascularization and limited angiogenic capacity (Gealekman et al., 2011). This hypoxic microenvironment promotes oxidative stress, adipocyte fibrosis, and cell death, which in turn trigger inflammatory gene expression in tissue-resident immune cells (Valenzuela et al., 2023), especially macrophages (Guria et al., 2023). These cells release pro-inflammatory cytokines, such as TNF-α and IL-6, thereby sustaining local inflammation and contributing to SLGCI (Valenzuela et al., 2023). Consistent with this, several observational studies have reported a positive association between VAT and circulating CRP levels (Forouhi et al., 2001; Saijo et al., 2004; Park et al., 2010; Tsuriya et al., 2011). Encouragingly, exercise—even as a standalone intervention—has been shown to reduce both subcutaneous (Yarizadeh et al., 2021) and visceral fat stores (Vissers et al., 2013; Sabag et al., 2017), with aerobic training, particularly at high intensities, appearing especially effective in targeting VAT (Ismail et al., 2012; Chen et al., 2024; Poon et al., 2024).

In addition to reducing VAT, exercise may also influence brown adipose tissue (BAT), a thermogenic and metabolically active tissue involved in energy homeostasis (Dong et al., 2023). Compared to white adipose tissue, BAT appears less prone to inflammatory signaling and may exert local anti-inflammatory effects (Omran and Christian, 2020). Preclinical studies suggest that exercise can enhance BAT activity, potentially improving metabolic and inflammatory profiles (Dong et al., 2023; Stroh and Stanford, 2023). However, findings from human studies remain inconsistent—with some RCTs, such as the ACTIBATE trial, showing no change in BAT volume or activation following 24 weeks of exercise in young sedentary adults (Martinez-Tellez et al., 2022). While promising, current evidence is insufficient to conclude that BAT activation is a key mechanism by which exercise impacts SLGCI or MDD.

Taken together regularly incorporating a variety of aerobic and resistance exercises into a routine aligned with ancestral activity patterns—while minimizing sedentary behavior—may provide a practical and physiologically-relevant strategy to reduce SLGCI and depressive symptoms through simultaneous improvements in physical fitness components, including body composition.

Further reinforcing this strategy, alternative exercise protocols such as short sprint interval training (sSIT) have also demonstrated promise. Recent findings by Ribeiro et al. (2024) showed that sSIT led to significant reductions in depressive symptoms, along with improvements in aerobic power, lower limb muscle power, body composition, and incidental PA levels in women with MDD—all achieved with less than 1 hour of total exercise over 2 weeks (Ribeiro et al., 2024). These preliminary findings underscore the potential of innovative, time-efficient exercise modalities that merit further investigation alongside more established exercise interventions for the treatment of MDD.

**Figure 1:**
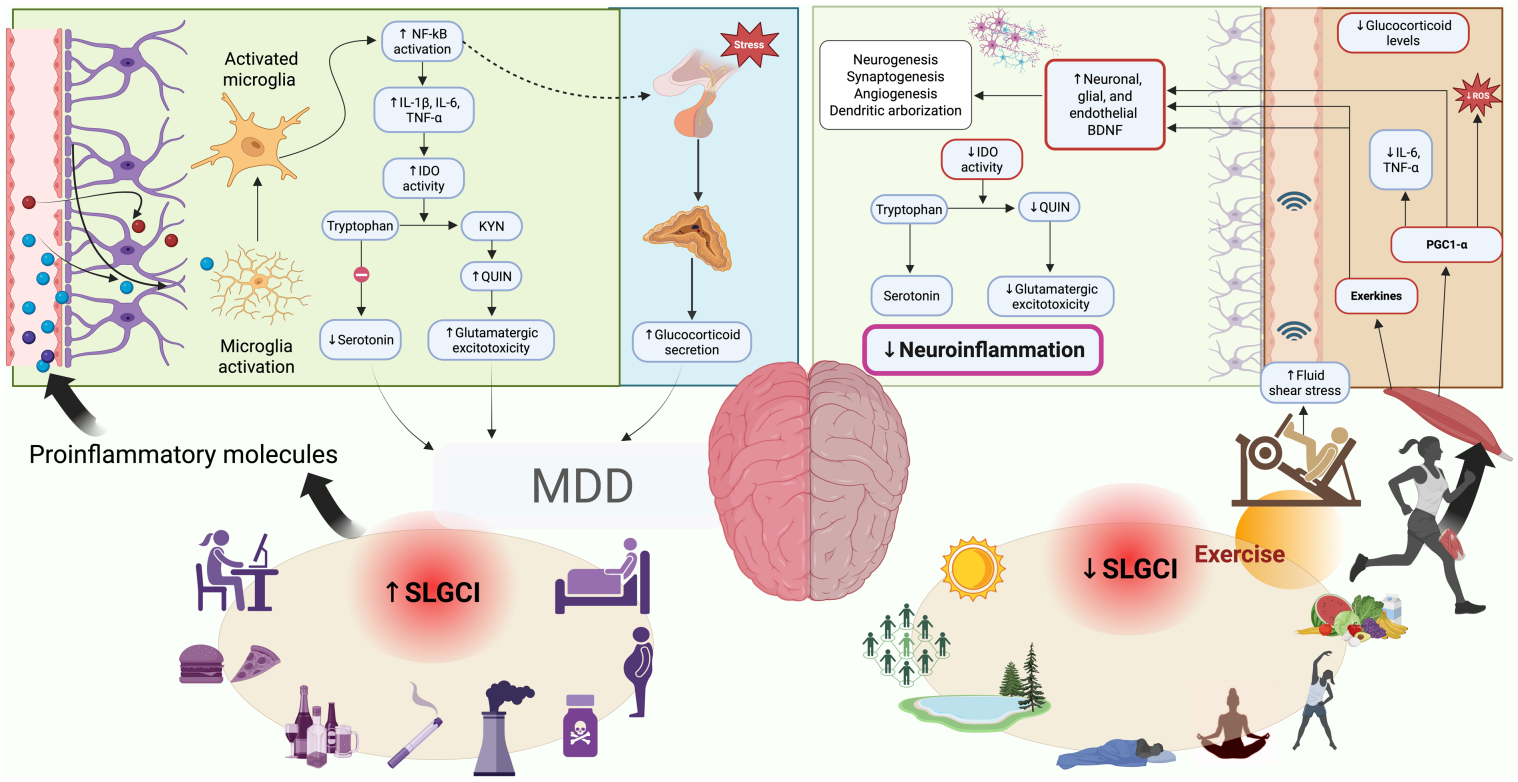
Conceptual framework illustrating how chronic inflammation contributes to Major Depressive Disorder (MDD), and how exercise and lifestyle factors may counteract it. On the left side, chronic low-grade inflammation (↑ SLGCI) is fueled by modern lifestyle factors such as poor diet, sedentary behavior, smoking, alcohol consumption, inadequate sleep, and psychosocial stress. This leads to increased systemic levels of proinflammatory molecules (e.g., IL-1β, IL-6, TNF-α), which can cross the blood–brain barrier and activate microglia. Activated microglia release additional cytokines and neurotoxic metabolites (e.g., quinolinic acid) via the kynurenine pathway, contributing to neuroinflammation, impaired serotonin signaling, glutamatergic excitotoxicity, reduced BDNF levels, and ultimately, the development of MDD or depressive symptoms. On the right side, regular exercise and healthy lifestyle habits—such as physical activity in natural environments, social interaction, adequate sunlight exposure, stress management, and a nutrient-rich diet—are associated with reduced systemic inflammation (↓ SLGCI). Exercise promotes anti-inflammatory effects through various pathways, including shear stress-induced production of exerkines (e.g., IL-6 with anti-inflammatory action, BDNF), improved mitochondrial function, and modulation of immune responses. These adaptations help decrease peripheral and central inflammation, enhance neuroplasticity, and alleviate depressive symptoms. BDNF, Brain-derived neurotrophic factor; IDO, indoleamine 2,3-dioxygenase; KYN, Kynurenine; MDD, Major Depressive Disorder. NF-κB, Nuclear Factor kappa-light-chain-enhancer of activated B cell; PGC1-α, Peroxisome proliferator-activated receptor gamma coactivator 1-alpha; QUIN, Quinolinic acid; SLGCI, Systemic low-grade chronic inflammation. Created with BioRender.com.

Figure 1 provides an overview of the proposed mechanisms linking exercise to reductions in inflammation and depressive symptoms.

## 6 Limitations and future perspectives

The approach proposed in this narrative review is not without limitations. Our framework is informed by a diverse body of evidence, including observational, mechanistic, and interventional studies—such as RCTs—examining the effects of PA and exercise on SLGCI and depressive symptoms. However, many of the specific associations discussed remain correlational, particularly regarding the interaction between evolutionary mismatches, SLGCI, and depression. Moreover, the potential role of other associated pathophysiological processes—such as gut microbiota dysbiosis (Van Baalen et al., 2025)—should not be overlooked, although they fall outside the scope of the present review.

Furthermore, while our model draws on ancestral activity patterns to inform modern interventions, these patterns are inferred from archeological data and ethnographic studies of contemporary hunter-gatherer populations who are themselves influenced by modern environments. Accordingly, although it is conceptually sound to align PA and exercise strategies with evolutionary insights, empirical testing of these hypotheses in humans remains a challenge. Nevertheless, targeted and well-designed RCTs can assess the effects of specific exercise modalities and training loads—particularly when embedded within lifestyle interventions—on neuroinflammatory and depressive outcomes.

Lifestyle change is inherently complex and nonlinear, and its success depends on a constellation of behavioral, environmental, and individual factors. While *Homo sapiens* may have partially adapted to more sedentary living since the Neolithic era, the evolutionary argument for an active lifestyle remains compelling. Still, generalizations based on ancestral patterns may not apply universally. Therefore, exercise-based interventions should be tailored to the individual’s physiological, psychological, and social context, ideally through a holistic strategy that targets priority lifestyle factors.

Importantly, humans did not evolve to “exercise” as a discrete activity (MacDonald et al., 2025), but to remain consistently active as part of daily life (Boullosa et al., 2013; Fiuza-Luces et al., 2018). Thus, lifestyle interventions should aim to identify the optimal combination of PA levels, reduced sedentary time, and intentional exercise that promotes long-term adherence through positive affective experiences. Future studies should explore how different exercise interventions—integrated within realistic, sustainable lifestyle strategies—can best modulate chronic inflammation and depressive symptoms across diverse populations and clinical contexts.

## 7 Conclusion

There is growing recognition that MDD is intricately connected to SLGCI, a condition driven and exacerbated by modern lifestyle factors that deviate from ancestral patterns of PA and environmental exposure. PA and, more specifically, exercise offer a robust, evidence-based intervention for modulating inflammation and improving depressive symptoms. Viewed through an evolutionary lens, aligning exercise patterns with those of our hunter-gatherer ancestors—characterized by regular low-to-moderate activities interspersed with occasional high-intensity efforts—emerges as a promising therapeutic strategy. This approach not only addresses some of the root causes of MDD but also provides broader benefits for general health and physiological resilience.

This narrative review highlights the dual role of PA and exercise as both preventive and therapeutic modalities for MDD, targeting key mechanisms such as SLGCI and neuroinflammation. While current evidence is encouraging, future research should focus on refining exercise protocols to maximize their efficacy, particularly for individuals with diverse backgrounds or comorbid conditions. Findings from the overtraining literature suggest that exercise may follow an optimal dose–response curve, in which excessively high levels could be counterproductive. In parallel, understanding the long-term effects of exercise on both mental and physical health in MDD populations remains a critical research priority.

Adopting a holistic perspective that integrates conventional treatment (e.g., anti-depressant medications) with exercise and other lifestyle modifications—including improved sleep hygiene, stress management, and dietary interventions—may offer the most comprehensive and sustainable approach for mitigating the global burden of MDD. By bridging ancestral behavioral patterns with contemporary science, exercise can reclaim its place as a cornerstone of mental health care, offering accessible, safe, and effective support for individuals worldwide.
